# The miRNA Pull Out Assay as a Method to Validate the miR-28-5p Targets Identified in Other Tumor Contexts in Prostate Cancer

**DOI:** 10.1155/2017/5214806

**Published:** 2017-09-20

**Authors:** Milena Rizzo, Gabriele Berti, Francesco Russo, Monica Evangelista, Marco Pellegrini, Giuseppe Rainaldi

**Affiliations:** ^1^Non-coding RNA Laboratory, Institute of Clinical Physiology (IFC), CNR, Via Moruzzi 1, 56124 Pisa, Italy; ^2^Tuscan Tumor Institute (ITT), Via Alderotti 26/N, 50139 Firenze, Italy; ^3^Instute of Life Science, Scuola Superiore Sant'Anna, Piazza Martiri delle Libertà 33, 56127 Pisa, Italy; ^4^Laboratory of Integrative Systems Medicine (LISM), Institute of Informatics and Telematics (IIT) and Institute of Clinical Physiology (IFC), CNR, Via Moruzzi 1, 56124 Pisa, Italy; ^5^Novo Nordisk Foundation Center for Protein Research, Faculty of Health and Medical Sciences, University of Copenhagen, Blegdamsvej 3b, 2200 Copenhagen, Denmark

## Abstract

miR-28-5p is an intragenic miRNA which is underexpressed in several tumor types showing a tumor suppressor (TS) activity. Routinely, the known miR-28-5p targets are validated in specific tumor contexts but it is unclear whether these targets are also being regulated in other tumor types. To this end, we adopted the miRNA pull out assay to capture the miR-28-5p targets in DU-145 prostate cancer (PCa) cells. Firstly, we demonstrated that miR-28-5p acts as a TS-miRNA in PCa, affecting cell proliferation, survival, and apoptosis. Secondly, we evaluated the enrichment of the 10 validated miR-28-5p targets in the pull out sample. We showed that E2F6, TEX-261, MAPK1, MPL, N4BP1, and RAP1B but not BAG1, OTUB1, MAD2L1, and p21 were significantly enriched, suggesting that not all the miR-28-5p targets are regulated by this miRNA in PCa. We then verified whether the miR-28-5p-interacting targets were regulated by this miRNA. We selected E2F6, the most enriched target in the pull out sample, and demonstrated that miR-28-5p downregulated E2F6 at the protein level suggesting that our approach was effective. In general terms, these findings support the miRNA pull out assay as a useful method to identify context-specific miRNA targets.

## 1. Introduction

It is well known that the deregulation of miRNA expression is one of the causes or contributory causes of cancer development. miRNAs may act as tumor suppressors (TS), oncogenes, or both depending on the tumor context [1]. miR-28-5p is an intragenic miRNA downregulated in several tumor types, such as hepatocellular carcinoma [2], renal cell carcinoma [3], natural killer/T-cell lymphoma [4], B-cell lymphoma [5], colorectal cancer (CRC) [6], and CRC liver metastasis [7, 8], although in some cases, an increased expression level of miR-28-5p has been observed (ovarian, esophageal, and cervical cancer) [9–11]. Most of the papers regarding the role of miR-28-5p in tumors suggested a prevalent tumor suppressor activity of this miRNA *in vitro* [2, 3, 5, 6, 12]. Very recently, it has been demonstrated that the miR-28-5p reexpression in xenograft models of Burkitt (BL) and diffuse large B-cell lymphoma (DLBCL) as well as in a BL murine model blocked tumor growth, opening the way to miR-28-5p-based replacement therapy as a novel therapeutic strategy for these diseases [13].

The molecular targets through which miR-28-5p exerts its anti- or proproliferative role are only partially known. For example, miR-28-5p reduced cell growth and migration in hepatocellular carcinoma [2] and in CRC [6] cells inhibiting the expression of IGF-1, CCND1, and HOXB3 genes. In addition, miR-28-5p acted as a TS-miRNA in renal cell carcinoma by directly repressing the expression of RAP1B [3] and in B-cell lymphoma by directly inhibiting BAG1 expression, a gene involved in the MAP-kinase pathway regulation [5].

To date, there are no data either on the role of miR-28-5p in prostate cancer (PCa) or on the targets regulated by this miRNA in PCa cells. In this work, we evaluated whether the miR-28-5p targets validated in other types of tumors were regulated in PCa cells using the miRNA pull out assay, a technique that allows the isolation of all the targets of a given miRNA in specific biological contexts. We demonstrated that miR-28-5p exerted TS activity in PCa cells and that not all validated miR-28-5p targets are regulated by this miRNA in the PCa context.

## 2. Materials and Methods

### 2.1. Cell and Culture Conditions

DU-145 and A-549 cells were grown in RPMI Medium 1640 (EuroClone) whereas PC-3 cells were grown in HAM's medium (EuroClone) and MCF-7 cells in DMEM low glucose (EuroClone). 10% FBS (fetal bovine serum, EuroClone), 1% penicillin/streptomycin (2 mM, EuroClone), and 1% L-glutamine (2 mM, Sigma-Aldrich) were added to the medium. The cells were incubated at 37°C in a humidified atmosphere containing 5% CO_2_.

### 2.2. Transfection

Transient transfections of double-stranded miRNA mimics (miR-28a-5p) or negative control (miR-NC) (GenePharma) in DU-145 cells were carried out using Lipofectamine 2000 (Thermo Fisher): 1.5 × 10^5^ cells were seeded in P30 dishes and after 48 hours, cells were transfected with miRNA mimic using 10 *μ*l of Lipofectamine according to the protocol provided by the manufacturer. The suspension of the transfected cells was used for cellular and molecular assays.

### 2.3. Cell Proliferation

1 × 10^5^ cells were seeded in a series of 30 mm diameter dishes and grown for 96 hours. At 24-hour intervals, cells were collected and counted.

### 2.4. Cell Cycle Analysis

Cell cycle analysis was performed as follows: 5 × 10^5^ cells were fixed with 95% cold ethanol and labelled with 300 *μ*l of 50 *μ*l/ml propidium iodide (Sigma-Aldrich) solution. After overnight incubation at 4°C, the cell cycle analysis was performed with Accuri™ C6 flow cytometer (BD Biosciences). Using specific software supplied with the instrument, the percentage of cells in each phase of the cell cycle was determined considering the parameters SSC-H/FL2-A.

### 2.5. Survival Assay

Survival was measured as follows: cells were collected and seeded at cell density of 200 cells/60 mm diameter culture dish to allow colony formation. After 10–12 days, dishes were stained with 0.1% CV and the ratio (number of colonies/number of seeded cells) was used to calculate the fraction of surviving cells.

### 2.6. Apoptosis Assay

Apoptosis was measured as follows: 1 × 10^6^ cells were suspended in 300 *μ*l Binding Buffer 1X and left at room temperature for 15 minutes. Thereafter, cell labelling was done according to the kit Annexin V-FITC. Cells were then passed through flow cytometer BD Accuri C6 (BD Biosciences) and analyzed using FL3-H/FL1-H parameters.

### 2.7. miRNA Pull Out Assay

The miRNA pull out assay was performed as described in Rizzo et al. [14]. Briefly, DU-145 cells were transfected using Lipofectamine 2000 (Thermo Fisher) with 60 nM of either miR-28a-5p duplex (ds-miR-28a_CT_) or a mix of 3′ biotin-tagged miR-28a-5p 8tU (nucleotide 8 was a thiouridine) and miR-28a-5p 18tU duplexes (ds-miR-28a_BIO_). The day after transfection, cells were irradiated with UV (365 nm, 2 J/cm^2^) using Bio-Link crosslinking (BLX) (Ambrose Lourmat) and total RNA extracted adding TRIzol reagent (Thermo Fisher) directly to adherent cells and following the instructions provided by the manufacturer. 15 *μ*g of RNA was incubated for 4 hours at 4°C with 100 *μ*l of streptavidin-conjugated beads (Streptavidin Sepharose High Performance, GE Healthcare), and the RNA complexed with the beads recovered using the Trizol protocol. We performed three biological replicates obtaining three miR-28_CT_ (miR-28 control) and three miR-28_BIO_ (miR-28) pull out samples.

### 2.8. Quantification of miRNAs and mRNAs (qRT-PCR)

Total RNA was extracted from 1 × 10^6^ cells using the miRNeasy mini kit following the manufacturer's recommendations. 1 *μ*g of total RNA was retrotranscribed using either the miScript II RT kit (Qiagen) or the QuantiTect Reverse Transcription Kit (Qiagen) for the miRNA or the mRNA quantification, respectively. The reverse transcription was performed following the manufacturer's instructions. miRNAs and the mRNAs were quantified with Rotor-Gene Q 2plex (Qiagen), using the miScript SYBR Green PCR Kit (Qiagen) and the SsoAdvanced™ SYBR® Green Supermix (Bio-Rad), respectively, according to the manufacturer protocols. The relative quantification was performed using the Rotor-Gene Q software, normalizing to the internal controls (U6, SNORD55, and SNORD110 for miRNA and GAPDH, ACTB, and HPRT for mRNA). The relative miR-28a-5p expression level in tumor cell lines was evaluated with respect to the normal cell RNA (FirstChoice Human Total RNA, Ambion). All reactions were performed in triplicate, and the results are the mean of three biological replicates.

### 2.9. Western Blot Analysis

Proteins were extracted from cell pellets using lysis buffer (1 M Tris HCl pH 8, Triton X-100 1%, and Na deoxycholate 0.25%) with the addition of PMSF 1 mM. The proteins were then quantified colorimetrically using the BioRad protein Assay Reagent (Bio-Rad). Absorbance was measured at 595 nm with ChroMate microplate reader (Awareness Technology). The proteins were separated on polyacrylamide gels SDS-PAGE (10%, gel precast Mini-PROTEAN® TGX Stain-Free™, Bio-Rad) and transferred to 0.2 *μ*m nitrocellulose membranes by electro blotting using the Trans-Blot Turbo Blotting System (Bio-Rad). The resulting blots were blocked with 5% nonfat dry milk solution in TBST. Anti-GAPDH (Cell Signaling) (1 : 20000), anti-E2F6 (Santa Cruz Biotechnology) (1 : 500), and PARP-1 (Santa Cruz Biotechnology) (1 : 500) primary antibodies were used. Incubation was performed overnight at 4°C, and bands were revealed after incubation with the recommended secondary antibody coupled to peroxidase using ECL (GE Healthcare). Scanned images were quantified using ImageJ software and normalized to GAPDH.

### 2.10. Statistical Analyses

Results are expressed as mean SD of at least three independent experiments, and data are analyzed using Student's *t*-test (^∗^*P* < 0.05, ^∗∗^*P* < 0.01, ^∗∗∗^*P* < 0.001, and ^∗∗∗∗^*P* < 0.0001).

## 3. Results

### 3.1. miR-28-5p Exerted a TS Activity in PCa Cells

**Figure 1:**
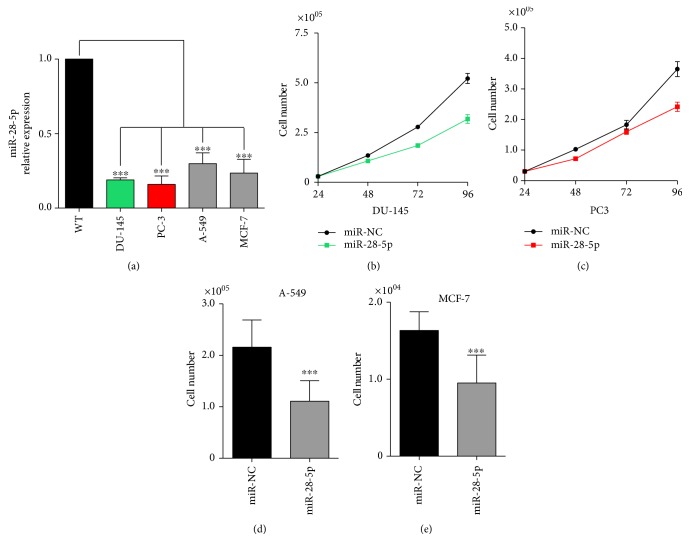
miR-28-5p expression and effect on tumor cells. (a) Analysis of the miR-28-5p expression level with qRT-PCR in prostate (PC-3 and DU-145), lung (A-549), and breast (MCF-7) cancer cell lines compared to the normal cell RNA. Cell proliferation of DU-145 (b), PC-3 (c), A-549 (d), and MCF-7 (e) cells at different time points or at 96 hours after the miR-28-5p reexpression. ^∗∗∗^*P* < 0.001, unpaired *t*-test.

To investigate the role of miR-28-5p in PCa, we first evaluated its expression in two PCa cell lines (DU-145 and PC-3) compared to normal cells (Figure 1(a)). We also evaluated the expression of miR-28-5p in lung (A-549) and breast (MCF-7) cancer cells, demonstrating that miR-28-5p was markedly downregulated in all the analyzed cancer cell lines.

In order to test whether miR-28-5p behaves as a TS in PCa cells, we first measured cell proliferation of DU-145 and PC-3 cells after miR-28-5p reexpression. Data showed a significant inhibition of cell proliferation of both PCa cell lines (Figures 1(b) and 1(c)). Similar results were obtained when the miR-28-5p was transfected in breast and colon cancer cell lines (Figures 1(d) and 1(e)).

**Figure 2:**
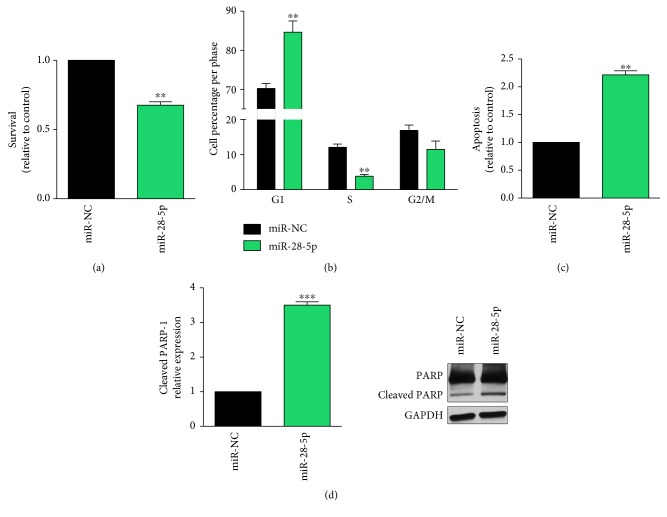
Effects of miR-28-5p reexpression on DU-145 cells. Cell survival (a) and cell cycle (b) in DU-145 cells after miR-28-5p reexpression. Apoptosis analysis measured with both annexin assay (c) and western blot of PARP-1 and cleaved PARP-1 (d) in miR-28-5p-transfected DU-145 cells. ^∗∗^*P* < 0.01 and ^∗∗∗^*P* < 0.001, unpaired *t*-test.

To further investigate the biological effects of the miR-28-5p in PCa, we checked the colony-forming ability (CFA) and the cell cycle after miRNA reexpression in DU-145 cells. Data showed that miR-28-5p reexpression resulted in both a significant reduction of CFA (Figure 2(a)) and a slight but significant increase of cells in G1 phase (Figure 2(b)), suggesting that the proliferation was negatively affected. Finally, we demonstrated that the miR-28-5p reexpression increased apoptosis in DU-145 cells (Figures 2(c) and 2(d)). Overall data suggested that the miR-28-5p acts as a TS-miRNA in PCa, probably by regulating key pathways involved not only in tumor cell proliferation but also in tumor cell survival.

### 3.2. Some Validated miR-28-5p Targets Interacted with miR-28-5p in PCa

To investigate which targets were regulated by miR-28-5p in PCa, we transfected this miRNA in DU-145 cells and the miRNA pull out assay was performed [14]. This technique allowed the capture and the isolation of the miR-28-5p/target complexes using a biotinylated version of miR-28-5p. We considered the miR-28-5p targets deposited in miRTarBase (http://mirtarbase.mbc.nctu.edu.tw), in particular, the ones validated with the luciferase reporter assay (Table 1).

**Figure 3:**
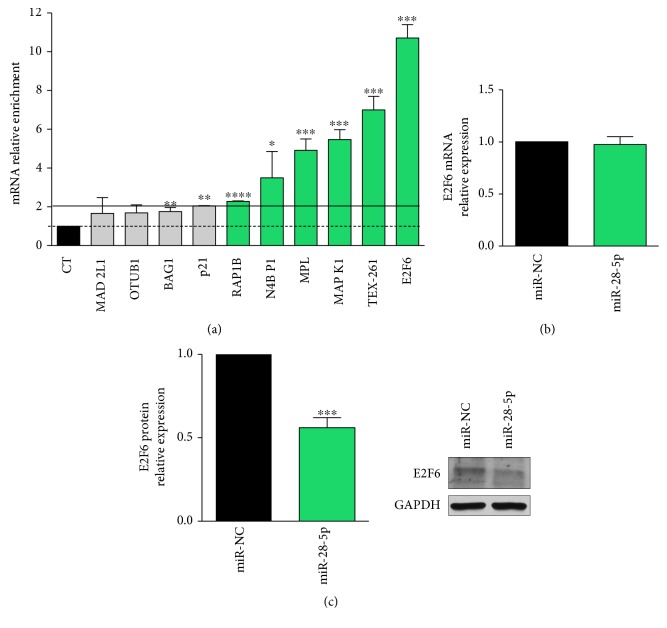
miR-28-5p target validation. (a) miR-28-5p-validated target enrichment quantified by qRT-PCR in miR-28-5p pull out sample compared to miR-28-5p control pull out sample (CT). E2F6 mRNA (b) and protein (c) quantification in DU-145 cells transfected with miR-28-5p or miR-NC. ^∗^*P* < 0.05, ^∗∗^*P* < 0.01, ^∗∗∗^*P* < 0.001, and ^∗∗∗∗^*P* < 0.0001 unpaired *t*-test.

Using qRT-PCR, we checked the enrichment of these targets in the pool of miR-28-5p-captured targets (miR-28 pull out sample) and found that RAP1B, N4BP1, MPL, MAPK1, TEX-261, and E2F6 were enriched by more than 2-fold in the miR-28-5p pull out sample (Figure 3(a)). These results suggested that not all the miR-28-5p-validated targets interact and, as a consequence, may be regulated by miR-28-5p in PCa cells.

To verify whether the enrichment of the selected targets in the miR-28-5p pull out sample was indicative of the miRNA regulatory function, we selected the most enriched one, that is, E2F6, and determined its expression after the miR-28-5p reexpression in DU-145 cells. We demonstrated that E2F6 was inhibited by miR-28-5p reexpression only at the protein level (Figures 3(b) and 3(c)), indicating that E2F6 was regulated by miR-28-5p in PCa.

## 4. Discussion

miRNAs are key inhibitors of gene expression that play a pivotal role in tumor development and progression affecting genes and pathways involved in all the hallmarks of cancer [17, 18]. By regulating oncogenes or TS genes, they can act, respectively, as a TS or oncogenes, although it is known that the role of a specific miRNA in cancer is not absolute but strongly related to the tumor context [1]. Therefore, it is not surprising that miR-28-5p may act as an oncogene (e.g., [9]) or as a TS (e.g., [13]), even though in most tumors, it showed an antiproliferative effect. It is possible that, depending on the expression level of the targets or on the effect of regulators which interfere with miRISC binding or function, not all the targets of a particular miRNA can be bound and regulated by the miRNA in a specific context [19, 20].

In this work, performing the miR-28-5p pull out assay, we have explored whether the known miR-28-5p targets, deposited in miRTarBase and validated with at least the luciferase reporter assay, interacted and were regulated by miR-28-5p in PCa. Under this strategy was that, among all miR-28-5p-validated targets, the ones that interact with miR-28-5p in PCa have higher chance to be regulated by the miRNA in this tumor. We first evaluated the miR-28-5p role in PCa, and we demonstrated for the first time that this miRNA is underexpressed in these cells and that its reexpression inhibited cell proliferation and survival. These data led us to conclude that miR-28-5p acted as a TS-miRNA in PCa. As it has been demonstrated that miR-28-5p negatively regulated genes involved in tumor cell growth in lung [12] as well as in colon rectal [6] cancer cells, we checked the expression level and the effects of miR-28-5p in lung and colon cancer cell lines as a positive control.

Using the miRNA pull out assay, we found that not all the validated miR-28-5p targets were enriched in the miR-28 pull out sample. Among the enriched targets that are more strongly associated with cancer (i.e., RAP1B, N4BP1, MAPK1, and E2F6), almost all are protumoral. Indeed, both MAPK1 and RAP1B, a Ras-related small GTP-binding protein that acts as GTPase in several signaling cascades, are proproliferative proteins involved as oncogenes in the development and progression of several tumor types (e.g., [21, 22]). In particular, it has been demonstrated that miR-28-5p suppressed cell proliferation and migration by directly inhibiting RAP1B in renal cell carcinoma [22]. These observations are consistent with a possible regulation by miR-28-5p, suggesting the utility of our approach to identify context-specific miRNA targets. In the same way, it has been reported that when PCa evolves from a benign to more aggressive stage, it becomes resistant to apoptosis due to the increased expression of antiapoptotic proteins [23] such as E2F6 [24]. Indeed, we demonstrated that E2F6, the most enriched target in the miR-28-5p pull out sample, was regulated by miR-28-5p at the posttranscriptional level. We can speculate that the target enrichment level in the miRNA pull out sample might facilitate the identification of the targets affected by the regulation of the miRNA. In addition, we also showed that the miR-28-5p reexpression induced apoptosis in DU-145 cells. Given that DU-145 cells (androgen independent PCa cell line) are an advanced PCa *in vitro* model, miR-28-5p reexpression may be taken into consideration as a novel therapeutic approach for PCa at this stage. Furthermore, we demonstrated that E2F6 is regulated by miR-28-5p in DU-145 cells; thus, it is conceivable that E2F6 could be one of the mediators of the apoptosis induced by miR-28-5p reexpression.

In conclusion, in this work, we demonstrated that the capture of the targets that interact with a given miRNA in a specific tumor is a suitable approach to identify the subset of targets that have a higher probability of being regulated by that miRNA in the context under evaluation. In the future, the identification of all the miR-28-5p targets (miR-28-5p targetome) could help to decipher the genes and pathways affected by the regulation of this miRNA in PCa.

## Figures and Tables

**Table 1:** miR-28-5p targets validated with gene reporter assay according to miRTarBase.

miR-28-5p target	Tumor type	Reference
p21	Choriocarcinoma cells	[15]
MPL	Myeloproliferative neoplasms	[16]
N4BP1	Myeloproliferative neoplasms/ovarian cancer	[9]
OTUB1	Myeloproliferative neoplasms	[16]
TEX-261	Myeloproliferative neoplasms	[16]
MAPK1	Myeloproliferative neoplasms	[16]
E2F6	Myeloproliferative neoplasms	[16]
MAD2L1	B-cell lymphomas	[5]
BAG1	B-cell lymphomas	[5]
RAP1B	B-cell lymphomas/renal cell carcinoma	[3, 5]
